# Static and Functional Hemodynamic Profiles of Women with Abnormal Uterine Artery Doppler at 22–24 Weeks of Gestation

**DOI:** 10.1371/journal.pone.0157916

**Published:** 2016-06-16

**Authors:** Åse Vårtun, Kari Flo, Christian Widnes, Ganesh Acharya

**Affiliations:** 1 Women’s Health and Perinatology Research Group, Department of Clinical Medicine, Faculty of Health Sciences, UiT- The Arctic University of Norway and Department of Obstetrics and Gynaecology University Hospital of Northern Norway, Tromsø, Norway; 2 Department of Clinical Sciences, Intervention and Technology, Karolinska Institute, Stockholm, Sweden; University of Southampton, UNITED KINGDOM

## Abstract

**Objective:**

To compare cardiac function, systemic hemodynamics and preload reserve of women with increased (cases) and normal (controls) uterine artery (UtA) pulsatility index (PI) at 22–24 weeks of gestation.

**Materials and Methods:**

A prospective cross-sectional study of 620 pregnant women. UtA blood flow velocities were measured using Doppler ultrasonography, and PI was calculated. Mean UtA PI ≥ 1.16 (90th percentile) was considered abnormal. Maternal hemodynamics was investigated at baseline and during passive leg raising (PLR) using impedance cardiography (ICG). Preload reserve was defined as percent increase in stroke volume (SV) 90 seconds after passive leg raising compared to baseline.

**Results:**

Mean UtA PI was 1.49 among cases (n = 63) and 0.76 among controls (n = 557) (p < 0.0001). Eighteen (28.6%) cases and 53 (9.5%) controls developed pregnancy complications (p <0.0001). The mean arterial pressure and systemic vascular resistance were 83 mmHg and 1098.89±293.87 dyne s/cm^5^ among cases and 79 mmHg and 1023.95±213.83 dyne s/cm^5^ among controls (p = 0.007 and p = 0.012, respectively). Heart rate, SV and cardiac output were not different between the groups. Both cases and controls responded with a small (4–5%) increase in SV in response to PLR, but the cardiac output remained unchanged. The preload reserve was not significantly different between two groups.

**Conclusion:**

Pregnant women with abnormal UtA PI had higher blood pressure and systemic vascular resistance, but similar functional hemodynamic profile at 22–24 weeks compared to controls. Further studies are needed to clarify whether functional hemodynamic assessment using ICG can be useful in predicting pregnancy complications.

## Introduction

Pregnancy is characterized by profound alterations in maternal systemic hemodynamics and cardiac function [1, 2]. The systemic vascular resistance (SVR) decreases as early as five weeks of gestation, followed by a significant increase in circulating blood volume and cardiac output (CO) [3–6]. Increased blood volume and red cell mass contribute to an increase in preload and the fall in SVR leads to decreased afterload [7]. These alterations are largely completed during the first half of pregnancy [3, 5, 8], and the cardiovascular adaption to pregnancy results in cardiac remodeling and increased left ventricular mass leading to altered systolic and diastolic performance [3, 9–11]. Changes in cardiovascular function caused by altered preload and afterload may be different among women developing pregnancy complications, such as preeclampsia, compared to those who remain healthy [12–14].

Increased pulsatility index (PI) and presence of early diastolic notch in the uterine artery (UtA) Doppler velocity waveforms have been used to identify women at risk of developing preeclampsia [15, 16] and other pregnancy complications [17]. However, UtA Doppler has not proven to be an effective screening tool in low-risk pregnancies. Using echocardiography, asymptomatic cardiac diastolic dysfunction has been shown to be present at mid-gestation (20–23 weeks) in women who subsequently develop preterm preeclampsia [18]. Therefore, evaluation of maternal cardiac function together with UtA Doppler might improve in the prediction of pregnancy complications. Furthermore, dynamic functional assessment of maternal cardiovascular function seems to provide more valuable information than the conventional static measurements [19–21]. Changes in cardiovascular function in response to a transient increase in volume load caused by passive leg raising (PLR) has been widely used as a method of functional hemodynamic assessment, especially in intensive care units [22].

We hypothesized that pregnant women with increased UtA PI have a different hemodynamic profile and an altered response to a transient volume load compared to controls. The specific aim of this study was to investigate the differences in static and functional hemodynamic profiles of women with high and normal UtA PI.

## Materials and Methods

### Study population

This was a prospective cross-sectional study of 620 pregnant women at 22–24 weeks of gestation. Pregnant women attending the antenatal clinic for routine antenatal ultrasound screening at 17–20 weeks of gestation were informed about the study and invited to participate. Those who gave informed written consent were recruited consecutively. Inclusion criteria were age >18 years, live singleton pregnancy with and no obvious fetal abnormality detected on ultrasound scan. Exclusion criteria were, diagnosis of a multiple pregnancy, and inability to communicate in Norwegian or English.

The study was performed during 2006–2013. PLR was added to the research protocol starting 2010. The participants were examined after approximately 8 hours of fasting, in a quiet room with temperature maintained at approximately 22°C between 8:00 to 12:00 hours. Height and weight was measured and used for calculation of body mass index (BMI) as: BMI = weight/height^2^. Body surface area (BSA) was calculated using the Du Bois formula as: BSA (m^2^) = 0.007184 x Height ^0.725^ x Weight ^0.425^ [23].

Information about the course and outcome of pregnancy including delivery data was obtained from the electronic medical records. Gestational hypertension was defined as blood pressure ≥140/90 mmHg in a previously normotensive women in the absence of proteinuria, and pre-eclampsia as blood pressure ≥140/90 mmHg and proteinuria of ≥ 300 mg/24 hours (or 1+ or more on a spot urine dipstick test) or HELLP ((hemolysis, elevated liver enzymes, low platelets) syndrome occurring after 20 weeks of gestation. Gestational diabetes was defined as a two-hour glucose concentration of ≥ 7.8–≤11.1 mmol/L after a 75g oral glucose tolerance test.

### Impedance cardiography

Maternal systemic hemodynamics and cardiac function was investigated using non-invasive impedance cardiography (ICG) (Philips Medical Systems, Androver, MA, USA). The use of ICG in pregnant women has been described previously [2, 20]. In short four pairs of sensors were used; two pairs applied vertically on each side at the axillary line of thorax and the other two pairs placed vertically on each side of the neck. A sphygmomanometer cuff was placed on the left arm and connected to the ICG instrument for blood pressure measurement. Central venous pressure (CVP) and pulmonary artery occlusion pressure (PAOP) were preset to 4 and 8 mmHg, respectively. Varibles describing systemic hemodynamics, i.e. cardiac output (CO), cardiac index (CI), stroke volume (SV), stroke index (SI), systemic vascular resistance (SVR) and systemic vascular resistance index (SVRI), as well as cardiac contractility and work, i.e. acceleration index (ACI), velocity index (VI), pre-ejection period (PEP), left ventricular ejection time (LVET), systolic time ratio (STR) calculated as (PEP/LVET) x 100%, and left ventricular cardiac work index (LCWI) were continuously recorded by ICG and displayed on screen. In addition, thoracic fluid content (TFC) was simultaneously recorded by ICG.

ACI is defined as the peak acceleration of blood flow from left ventricle into aorta, VI is the peak velocity of the systolic wave of aortic blood flow, PEP is the time from the beginning of electrical stimulation of the ventricles to the opening of the aortic valve (electrical systole), reflecting the pre-ejection period (isovolumetric ventricular contraction time), LVET is the time from the opening to the closing of the aortic valve (mechanical systole) and LCWI is the work performed by the left ventricle normalised for body surface area.

The measurements were performed in two different positions, at baseline with the women lying in a semi-recumbent position and after PLR as described previously [20]. Preload reserve was defined as percent increase in SV 90 seconds after PLR compared to baseline.

The women were told not to speak or move while measurements were conducted. Baseline measurements were recorded after 10 minutes of rest in a semi-recumbent position. Upper part of the bed was then lowered and the lower part of the bed was elevated to 45° electronically to achieve PLR. Hemodynamics measurements were recorded again at approximately 90 seconds after PLR. Cardiovascular response to PLR was expressed as percent change from the baseline value as. A single operator (ÅV) performed all ICG measurements under identical conditions.

### Doppler ultrasonography

Ultrasound examination was performed using an Acuson Sequoia 512 ultrasound system (Mountain View, CA,USA) with a 2.5–6 MHz curvilinear transducer. Two experienced sonographers (CW and KF) performed all examinations strictly adhering to the ALARA (as low as reasonably achievable) principle [24]. The pregnant woman was examined in a semi-recumbent position to avoid possible compression of inferior vena cava by the gravid uterus. Blood flow velocity waveforms were recorded from the left and right UtA proximal to the apparent crossover with the external iliac artery using color-directed pulse-wave Doppler as previously described [25]. Online measurements of velocities were obtained from four to six uniform Doppler velocity waveforms, and the average value of three successive cardiac cycles was recorded for analysis. UtA PI was calculated as: (peak systolic velocity—end-diastolic velocity)/time-averaged maximum velocity. The PI values from the left and right UtA were averaged and used for statistical analysis. The presence of a bilateral or unilateral diastolic notching was recorded.

### Statistical analysis

Data were analyzed using IBM SPSS statistics (SPSS software, version 22.0, Chicago, IL, USA). Women were divided into two groups based on the UtA PI. Those with a mean UtA PI ≥1.16 (above 90^th^ percentile for 22–24 weeks of gestation) were defined as cases and those with a mean UtA PI < 1.16 as controls.

Continuous variables are presented as mean ±SD. Discrete quantitative and categorical variables are presented as median (range) or n (%) as appropriate. Data distribution was evaluated by visual inspection of plots and assumption of normality was tested using Shapiro-Wilk test. We used parametric tests if the distribution of data did not deviate significantly from normality. Independent samples t-test was used for comparing cases and controls. Paired sample t-test was used for analysis of differences in hemodynamic variables measured at baseline and after passive leg raising within each group. Chi-squared test was applied for the analysis of differences in proportions between groups. Statistical significance was set as p < 0.05.

### Ethics statement

The study protocol was approved by the Regional Committee for Medical Research Ethics in North Norway (Ref.nr. 5.2005.1386 and 2010/586). All study participants gave informed written consent.

## Results

A total of 620 pregnant women with singleton pregnancy participated in the study. None of the participants were lost to follow up, and information on the course of pregnancy and perinatal outcome was available for all.

### Baseline characteristics and pregnancy outcome

The baseline characteristics of the study population including neonatal outcome are presented in Table 1. There were no significant differences in age and body mass index (BMI) between the two groups. About half of the participants in both groups were nulliparous. Prevalence of bilateral notching in UtA Doppler waveform among cases was 23 (36.5%) compared with 4 (0.7%) among controls (p <0.0001).

**Table 1:** Baseline characteristics of the maternal study population and birth outcome of neonates of mothers with normal and high mean UtA PI.

Parameter	Normal UtA PI (n = 557)	High UtA PI (n = 63)	p-value*
**Maternal**			
Age (years)	30 (range 18–44)	30 (range 19–40)	0.364
Body mass index at first examination (Kg/m^2^) (20–24 weeks)	26.08 ± 4.02	27.35 ± 5.29	0.069
Mean arterial pressure at baseline, first examination (mmHg) (22–24 weeks)	79.29 ± 8.04	82.76 ± 10.06	0.010
Nulliparous	290 (52.1)	29 (46.0)	0.365
Bilateral notch (%)	4 (0.7)	23 (36.5)	<0.0001
Mean UtA PI	0.76 ± 0.18	1.49 ± 0.40	<0.0001
**Birth outcome**			
Cesarean section (%)	58 (10.4)	10 (15.9)	0.2008
Gestational age at birth (weeks)	40 (range 25–42)	38 (range 25–42)	0.002
Birth weight (g)	3567 ± 531	3063 ± 771	<0.0001
Placental weight (g)	625 ± 138	540 ± 149	<0.0001
5-minute Apgar score	10 (range 2–10)	10 (range 5–10)	0.883
Umbilical artery pH	7.23 ± 0.09	7.24 ± 0.06	0.554
Umbilical artery base excess (mmol/L)	-4.78 ± 3.52	-3.99 ± 2.60	0.124

Data presented as n (%), median (range) or mean ± SD as appropriate.

*represents the p-value for the difference between normal and high UtA PI groups (Independent samples t-test or Pearson chi-squared test).

Among cases, 18 women (28.6%) developed complications compared to 53 women (9.5%) among controls (p < 0.0001). In the case group, 11 women (17.5%) developed pre-eclampsia, and 5 of these in combination with intrauterine growth restriction (IUGR). Pre-eclampsia was diagnosed before 37 weeks in 8 women (72.7%) and before 34 weeks in 4 women (36.4%). In the case group, 4 women (6.4%) developed gestational hypertension and 2 women (3.2%) developed gestational diabetes.

Among controls, 21 women (3.8%) developed pre-eclampsia including 3 women with HELLP syndrome. Pre-eclampsia was diagnosed before 37 weeks in 2 women (9.5%) and before 34 weeks in 2 women (9.5%). Twenty women (3.6%) developed gestational hypertension and 8 women (1.4%) developed gestational diabetes. Two women (0.4%) had placental abruption at 32^+4^ and 41^+3^ weeks of gestation. Both of these women had uncomplicated pregnancy before the event. There were 2 intrauterine fetal deaths (0.4%) among controls. One occured in a nulliparous woman at 26^+6^ weeks of gestation (mean UtA PI = 0.97), and the other in a multiparous woman at 36^+5^ weeks of gestation (mean UtA PI = 0.81). No obvious causes for fetal demise were found at autopsy.

There were significant differences between cases and controls regarding gestational age at delivery, birth weight and placental weight but not for 5-minute Apgar score and umbilical artery blood gases and acid-base status (Table 1).

### Static hemodynamics

The results of maternal systemic hemodynamics and cardiac function at baseline measured by ICG are presented in Table 2. At baseline arterial blood pressures and systemic vascular resistance index (SVRI) were significantly higher among cases compared to controls, but the heart rate, SV and cardiac output were not different between the groups.

**Table 2:** Maternal systemic hemodynamics and cardiac function of the study group with normal and high UtA PI measured using impedance cardiography.

Parameter	Normal UtA PI (n = 557)	High UtA PI (n = 63)	p-value*
Cardiac output (L/min)	6.11 ± 1.32	6.02 ± 1.34	0.617
Cardiac index (L/min/m^2^)	3.35 ± 0.54	3.28 ± 0.58	0.370
Heart rate (/min)	79.30 ± 11.59	79.75 ± 13.05	0.777
Systolic blood pressure (mmHg)	101.73 ± 9.81	105.89 ± 12.69	0.014
Diastolic blood pressure(mmHg)	68.04 ± 7.75	71.43 ± 9.48	0.008
Mean arterial blood pressure (mmHg)	79.24 ± 7.97	82.94 ± 10.16	0.007
Systemic vascular resistance (dyne s/cm^5^)	1023.95 ± 213.83	1098.89 ± 293.87	0.012
Systemic vascular resistance index (dyne s m^2^/cm^5^)	1836.93 ± 331.15	1983.49 ± 473.96	0.020
Stroke volume (ml)	79.16 ± 16.31	78.17 ± 16.12	0.651
Stroke index (ml/ m^2^)	43.43 ± 6.40	42.65 ± 6.68	0.363
Thoracic fluid volume (1/kOhm)	27.78 ± 4.59	26.81 ± 4.63	0.113
Acceleration index (1/100s^2^)	126.35 ± 45.26	120.60 ± 44.72	0.339
Left ventricular work index (kg m/ m^2^)	3.45 ± 0.74	3.55 ± 0.82	0.341
Pre-ejection period (ms)	83.03 ± 15.76	85.11 ± 15.45	0.320
Left ventricular ejection time (ms)	263.05 ± 31.54	263.68 ± 31.62	0.880
Velocity index (1/1000s)	77.62 ± 22.77	73.62 ± 23.32	0.188
Systolic time ratio (%)	32.60 ± 7.86	33.22 ± 7.55	0.549

Data presented as mean ± SD.

*represents the p-value between normal and high UtA PI groups at baseline (Independent samples t-test).

### Functional hemodynamics

**Fig 1:**
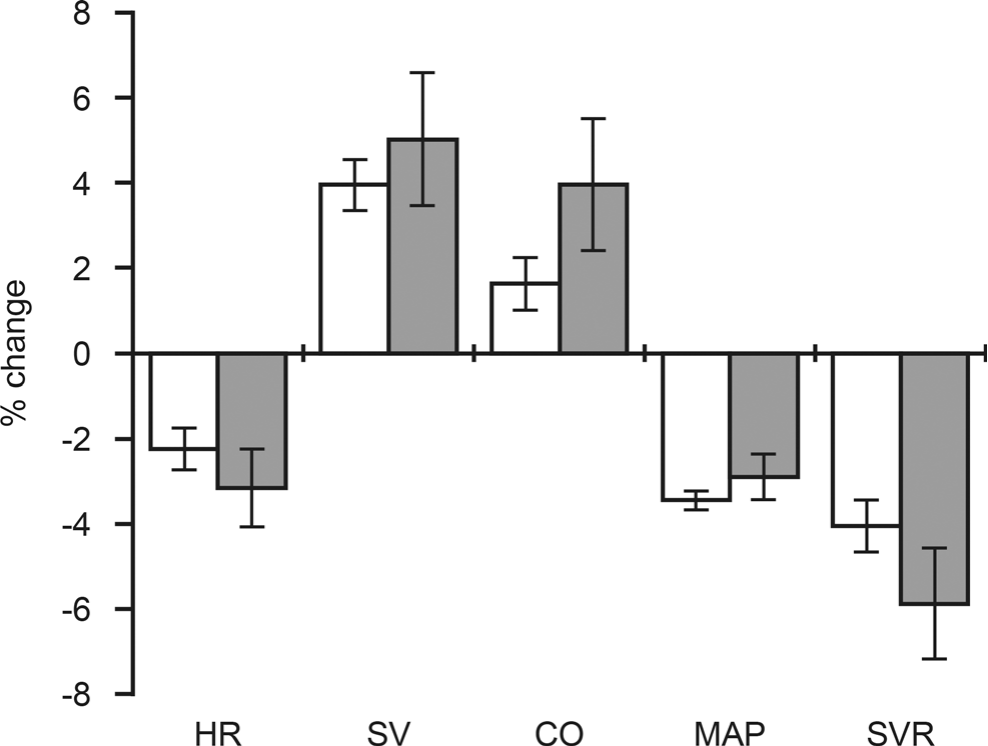
Differences in systemic maternal hemodynamics between pregnant women with normal and high (UtA PI). Data presented as mean ± standard error of mean (SEM). Maternal heart rate (HR), stroke volume (SV), cardiac output (CO), mean arterial pressure (MAP) and systemic vascular resistance (SVR). Percent change (∆%) representing the change from baseline to passive leg raising with normal (white bars) and high (grey bars) UtA PI.

Hemodynamic response to PLR was measured in 38 cases and 312 controls. The response following PLR described as percent change (∆%) in the measured parameters from baseline to PLR in each group is presented in Table 3. The direction and magnitude of change were similar for variables describing systemic blood flow and vascular resistance (Fig 1). A significant (p < 0.001) decreases in HR, MAP and SVR was observed both among the cases as well as controls during PLR. There was, a significant increase in SV among cases (p = 0.006) and controls (p < 0.001) following PLR, but the increase in CO was not significant in both groups.

**Table 3:** Hemodynamic parameters measured by impedance cardiography at baseline and 90 seconds after passive leg raising (PLR) in pregnant women with normal and high UtA PI.

	Normal UtA PI				High UtA PI				p-value*
Hemodynamic parameter	Baseline (n = 312)	PLR (n = 312)	% change	p-value^#^	Baseline (n = 38)	PLR (n = 38)	% change	p-value^#^	
SV (ml)	80.73 ± 17.03	83.33 ±16.71	3.95 ± 10.65	<0.001	80.37 ± 17.24	83.74 ± 16.53	5.02 ± 9.63	0.006	0.557
SI (ml/m^2^)	44.49 ± 6.64	45.90 ± 6.30	3.88 ± 10.68	<0.001	43.55 ± 7.34	45.50 ± 6.68	5.30 ± 9.82	0.004	0.437
CO (L/min)	6.26 ± 1.35	6.31 ± 1.26	1.63 ± 10.99	0.238	6.09 ± 1.55	6.26 ± 1.36	3.96 ± 9.59	0.092	0.212
CI (L/min/m^2^)	3.45 ± 0.56	3.48 ± 0.49	1.64 ± 11.05	0.223	3.29 ± 0.66	3.39 ± 0.53	4.13 ± 9.65	0.067	0.185
HR (/min)	79.91 ± 12.07	77.67 ± 10.47	-2.24 ± 8.61	<0.001	78.76 ± 14.02	75.84 ± 11.67	-3.16 ± 5.62	0.001	0.521
BPS (mm Hg)	101.26 ± 9.81	99.94 ± 9.70	-1.19 ± 4.39	< 0.001	107.58 ± 13.47	106.13 ± 11.34	-1.05 ± 4.34	0.056	0.860
BPD (mm Hg)	67.39 ± 7.87	63.84 ± 7.39	-5.09 ± 5.38	< 0.001	71.92 ± 10.68	69.08 ± 11.59	-4.08 ± 5.35	< 0.001	0.278
MAP (mm Hg)	78.66 ± 8.02	75.88 ± 7.83	-3.45 ± 3.89	< 0.001	83.84 ± 11.21	81.42 ± 11.25	-2.89 ± 3.35	< 0.001	0.390
SVR (dyne s/cm^5^)	993.34 ± 225.74	941.61 ± 178.96	-4.06 ± 10.67	<0.001	1116.32 ± 356.15	1040.53± 304.38	-5.87 ± 8.05	< 0.001	0.313
SVRI (dyne s m^2^/cm^5^)	1773.57 ± 359.98	1681.45 ± 266.07	-4.07 ± 10.67	< 0.001	2023.42 ± 571.93	1886.84 ± 487.50	-5.84 ± 8.22	< 0.001	0.322
TFC (1/kOhm)	29.64 ± 4.46	30.63 ± 4.40	3.73 ± 7.97	<0.001	28.61 ± 4.55	29.68 ± 4.45	4.11 ± 6.41	0.001	0.779
ACI (1/100 s^2^)	139.45 ± 49.84	133.68 ± 42.57	-.18 ± 26.06	0.001	127.21 ± 51.13	120.63 ± 46.80	-2.04 ± 19.98	0.089	0.671
LCWI (kg m/m^2^)	3.53 ± 0.73	3.41 ± 0.67	-2.43 ± 11.52	<0.001	3.59 ± 0.90	3.57 ± 0.76	.60 ± 10.89	0.722	0.125
PEP (ms)	80.64 ± 16.05	71.08 ± 13.90	-10.50 ± 15.32	< 0.001	84.55 ± 15.43	74.29 ± 12.91	-10.55 ± 16.25	< 0.001	0.985
LVET (ms)	259.42 ± 31.96	273.22 ± 33.59	6.35 ± 15.11	<0.001	265.71 ± 32.41	277.63 ± 33.98	5.71 ± 16.78	0.074	0.808
VI (1/1000 s)	85.66 ± 24.20	81.70 ± 20.47	-2.37 ± 16.94	<0.001	76.61 ± 26.40	75.66 ± 23.30	.78 ± 12.99	0.544	0.270
STR (%)	32.15 ± 8.41	26.73 ± 6.24	-14.06 ± 21.06	< 0.001	32.84 ± 7.96	27.58 ± 5.96	-12.38 ± 23.86	< 0.001	0.648

Data are presented as mean ± SD. % change is the difference between the measurements obtained at baseline and PLR in percent.

^**#**^represents the p-value for the difference between baseline and PLR (paired sample t-test) within normal and high UtA PI groups.

*represents the p-value for the difference in delta% values between normal UtA PI and high UtA PI groups (independent sample t-test). Stroke volume (SV), stroke index (SI), cardiac output (CO), cardiac index (CI), heart rate (HR), blood pressure systolic (BPS), blood pressure diastolic (BPD), mean arterial pressure (MAP), systemic vascular resistance (SVR), systemic vascular resistance index (SVRI), thoracic fluid content (TFC), acceleration index (ACI), left ventricular work index (LWCI), pre-ejection period (PEP), left-ventricular ejection time (LVET), velocity index (VI) and systolic time ratio (STR).

**Fig 2:**
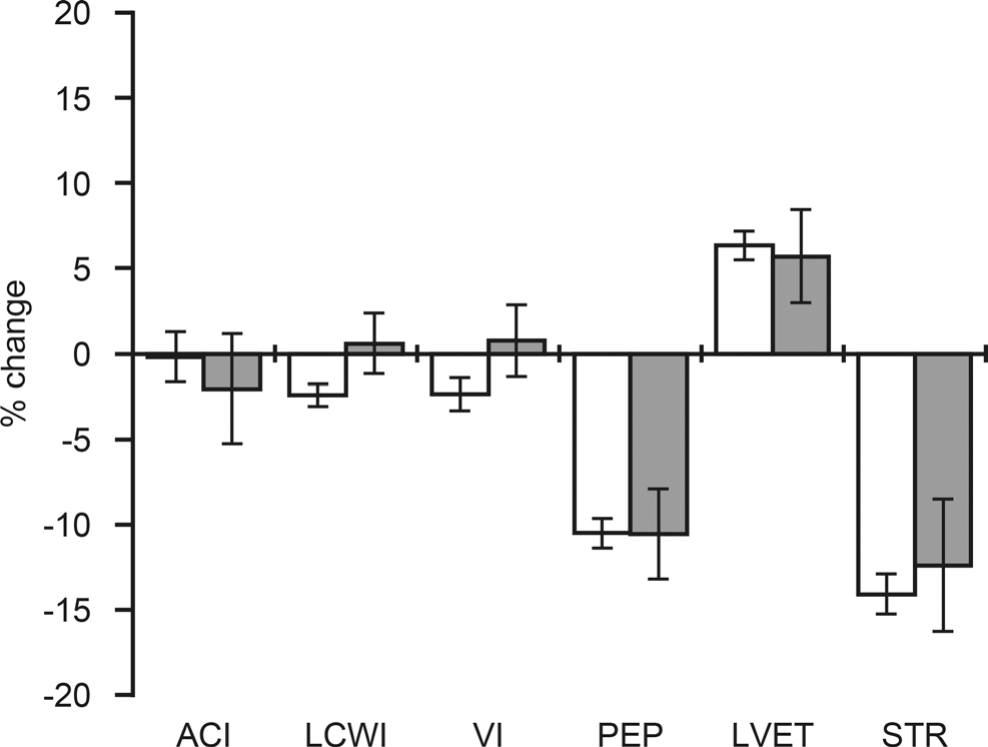
Differences in cardiac contractility and work between pregnant women with normal and high UtA PI. Data presented as mean ±standard error of mean (SEM). Percent change from baseline to passive leg raising. Normal (white bars) and high (grey bars) UtA PI. Acceleration index (ACI), left ventricular work index (LWCI), velocity index (VI), pre-ejection period (PEP), left-ventricular ejection time (LVET), and systolic time ratio (STR).

The direction and magnitude of changes in the parameters describing cardiac contractility and work in response to PLR are presented in Fig 2. PLR caused a significant (p <0.001) decrease in PEP and STR in both groups, but the changes in ACI, LVET, VI, and LCWI were significant only in control group (Table 3). The direction of change was opposite among cases and controls for VI and LCWI (Fig 2), but the magnitude of change was not significantly different between the groups.

Response to PLR was not different between cases and controls for any of the parameters of systemic hemodynamics and cardiac function investigated.

## Discussion

PLR causes a transient volume load resulting in significant changes in the majority of hemodynamic variables [20]. It has been widely used to predict fluid responsiveness in patients admitted to intensive care unit [26]. A recent study has shown that the women with severe pre-eclampsia associated with oliguria have reduced preload reserve [21]. As far as we know, hemodynamic response to PLR in women at risk of pregnancy complications has not been reported. Our study demonstrates that pregnant women with abnormal UtA PI have higher SVRI compared to controls, but their ability to respond to a small increase in preload caused by transient autotransfusion is preserved. We found similar preload reserve among women with high as well as normal UtA PI at 22–24 weeks of gestation.

A major strength of our study is that it was prospective, and we had a complete follow up on all recruited participants. There have been some concerns about the reproducibility and validity of ICG in pregnancy. However, ICG is a simple non-invasive method which is less operator dependent compared to echocardiography. We have previously found good repeatability of the measurements of SV, HR and CO and good agreement between measurements performed in semi-recumbent and left lateral positions using ICG [2, 27]. Other studies have validated ICG as a reliable, accurate and reproducible method reporting good agreement and correlation of ICG with echocardiography and thermodilution [28–33]. Furthermore, Burlingame et al have reported that the ICG has the ability of detecting small changes in maternal SV associated with position changes, whereas the echocardiography lacks the sensitivity to detect small changes in ejection fraction (EF) [30].

Investigation of the pregnant women was performed between 22 to 24 weeks of gestation. The placental circulation is well established by this gestational age and this is the period in gestation when UtA Doppler is most often performed in clinical settings. However, first trimester screening for pre-eclampsia using UtA Doppler in combination with other biochemical markers is gaining popularity in recent years. In this respect, it would have been interesting to study functional hemodynamic profile during 11–14 weeks of gestation. However, it was not feasible in our setting as first trimester ultrasound screening is not a routine in Norwegian national healthcare system. Another limitation of our study is that the sample size was insuffient to perform separate subgroup analyses comparing women who developed early and late-onset pre-eclampsia.

Ambient temperature can affect SVR. In our study, the room temperature was held at 22°C during all examinations. A study published in 2011 indicated a higher thermoneutral zone in pregnancy [34]. Another more recent study reported that the themoneutral zone for a clothed person at metabolic rest is between 17.5°C– 24.0°C [35]. What constitutes thermoneutral zone could be debatable, but we chose to keep the room temperature same throughout the whole study period and the measurements during baseline and PLR were performed at the same ambient temperature.

We found that approximately one third of women with high UtA PI developed complications during pregnancy and the prevalence of pre-eclampsia in our study population was 5.2%. Similar results have been reported by others [36–38]. Our findings of UtA notching were in accordance with Gómez et al [17] who recorded bilateral notches at 19–22 weeks in 32.8% of women who later developed pregnancy complications, and with Melchiorre et al [18] who found UtA notching in 23.7% of women with UtA PI >95^th^ percentile compared to 4.2% among controls at 20–23 weeks of gestation.

We found some differences in baseline maternal systemic hemodynamics, especially arterial blood pressure and SVR, between women with normal and abnormal mean UtA PI, but both groups responded similarly to PLR. Valensise et al [39] investigated 36 women with abnormal UtA RI (>0.58) and bilateral notch using Doppler echocardiography, and found that 41.7% of women who later developed complications had significantly higher MAP and SVR compared to normal outcome group. In another similar study, they found significantly higher MAP and SVR but lower CO in women developing early PE (<34 weeks of gestation) compared to controls, but the MAP was similar, SVR was lower, and CO was higher in the late PE group [12]. Khalil et al reported higher UtA PI and MAP in early pregnancy in women who later developed preterm pre-eclampsia compared to normal controls and the differences increased with gestational age [40].

In a previous study, we found that the healthy pregnant women at 22–24 weeks have small preload reserve as the SV increased only by a mean of 1.6 ml following PLR [20]. In the present study women with high UtA PI, demonstrated a slightly higher increase in SV (3.4 ml) compared to those with normal UtA PI (2.6 ml), but the difference was not significant. Heart rate and blood pressures decreased significantly in both groups following PLR. The thoracic fluid content (TFC) increased by approximately 4% after PLR indicating increased impedance related a shift in blood volume to supradiaphragmatic compartment from the lower part of the body.

The cardiac contractility variables, ACI and VI, are related to the systolic flow and aortic compliance, whereas the PEP and LVET respectively reflect the time required by the left ventricle to build up the pressure necessary to open the aortic valve and the duration of ventricular ejection. A reduction in ACI, VI and LVET and an increase in PEP, and LCWI may indicate reduced cardiac contractility. STR that represents the ratio of electrical to mechanical systole was decreased by PLR in both groups. The direction of change in these parameters suggests that the small increase in preload caused by PLR did not result in improved cardiac contractility. Although the magnitude of changes was not significantly different between groups, it was interesting to note that the LCWI and VI changed in opposite direction after PLR among cases (positive response) and controls (negative response) (Fig 2).

It has been shown that different states of hydration can modify the response to PLR [41], and that reduced plasma volume tends to increase the preload response. The observed differences between groups could be related to differences in volume state among women with high and normal UtA PI.

Investigators have used different cut-offs of mean UtA PI to predict pregnancy complications [15, 42], and all those values are higher than ours. We defined a mean UtA PI ≥90^th^ percentile at 22–24 weeks for our study population as abnormal. Fifty-three out of 557 women (9.5%) with a mean UtA PI below this cut-off developed complications during pregnancy. This could imply that the cut-off values for identifying women who are susceptible to complications later in pregnancy are set too high. Combining maternal risk factors and obstetric history, and UtA Doppler with the assessment of systemic hemodynamics and cardiac function using simple non-invasive methods might improve the sensitivity and specificity of predicting pregnancy complications [43].

## Conclusion

In summary, pregnant women with abnormal UtA PI had higher blood pressure and systemic vascular resistance index, but similar functional hemodynamic profile at 22–24 weeks compared to controls. Further studies are needed to clarify whether hemodynamic assessment using ICG can be useful in predicting pregnancy complications.
